# Supplementary material for: Effects of Different Na+ Concentrations on cAMP-Dependent Protein Kinase Activity in Postmortem Meat
Source: Foods. 2024 May 24;13(11):1647. doi: 10.3390/foods13111647 (PMC11171583; doi:10.3390/foods13111647)
Supplement: Supplementary file 1 [file foods-13-01647-s001.zip › foods-2992751-supplementary.pdf]

Table S1. The quality indicator results of muscle homogenate model/incubation model in different Na^+ concentrations at different time.

Index	*P*-value		
	Time	Na^+	Time ×Na^+
PKA activity (muscle homogenate model)	＜0.001	＜0.001	＜0.001
PKA activity (incubation model)	0.107	0. 027	＜0.001
ATP content (incubation model)	＜0.001	0.004	＜0.001
cAMP content	＜0.001	＜0.001	＜0.001
phosphorylation levels of sarcoplasmic protein	0.053	＜0.001	＜0.001
phosphorylation levels of myofibrillar protein	0.738	0.653	0.986
